# LCP1 knockdown in monocyte-derived macrophages: mitigating ischemic brain injury and shaping immune cell signaling and metabolism

**DOI:** 10.7150/thno.88678

**Published:** 2024-01-01

**Authors:** Yan Wang, Qianqian Yin, Decao Yang, Haojie Jin, Yang Yao, Jibing Song, Cuiying Liu, Yu Nie, Hao Yin, Wei Wang, Baohui Xu, Lixiang Xue, Xunming Ji, Xiaoyuan Chen, Heng Zhao

**Affiliations:** 1 Institute of Medical Innovation and Research, Peking University Third Hospital, Beijing, China.; 2 Department of Neurosurgery, Stanford University School of Medicine, 1201 Welch Road, MSLS Building, Stanford, USA.; 3 The Key Laboratory for Silviculture and Conservation of Ministry of Education, The College of forestry, Beijing Forestry University, Beijing, China.; 4 College of Chemistry, Beijing University of Chemical Technology, China.; 5 School of Nursing, Capital Medical University, Beijing, China.; 6 Fuwai Hospital, National Centre for Cardiovascular Diseases, Chinese Academy of Medical Sciences, Beijing, China.; 7 Organ Transplant Center, Shanghai Changzheng Hospital, Shanghai, China.; 8 Cell Transplantation and Gene Therapy Institute, The Third Xiang Ya Hospital, Central South University, Changsha, Hunan, China.; 9 Engineering and Technology Research Center for Xenotransplantation of Hunan Province, Changsha, China.; 10 Department of Surgery, Stanford University School of Medicine, 1201 Welch Road, MSLS Building, Stanford, USA.; 11 Beijing Institute of Brain Disorders, Capital Medical University, Beijing, China.; 12 Department of Diagnostic Radiology, Nanomedicine Translational Research Program, NUS Yong Loo Lin School of Medicine, National University of Singapore, Singapore.

**Keywords:** Stroke, Monocytes, Macrophages, Lipid Metabolism, Neuroinflammation, Immunodepression

## Abstract

**Rationale:** Ischemic stroke poses a significant health burden with limited treatment options. Lymphocyte Cytosolic Protein 1 (LCP1) facilitates cell migration and immune responses by aiding in actin polymerization, cytoskeletal rearrangements, and phagocytosis. We have demonstrated that the long non-coding RNA (lncRNA) Maclpil silencing in monocyte-derived macrophages (MoDMs) led to LCP1 inhibition, reducing ischemic brain damage. However, the role of LCP1 of MoDMs in ischemic stroke remains unknown.

**Methods and Results:** We investigated the impact of LCP1 on ischemic brain injury and immune cell signaling and metabolism. We found that knockdown of LCP1 in MoDMs demonstrated robust protection against ischemic infarction and improved neurological behaviors in mice. Utilizing the high-dimensional CyTOF technique, we demonstrated that knocking down LCP1 in MoDMs led to a reduction in neuroinflammation and attenuation of lymphopenia, which is linked to immunodepression. It also showed altered immune cell signaling by modulating the phosphorylation levels of key kinases and transcription factors, including p-PLCg2, p-ERK1/2, p-EGFR, p-AKT, and p4E-BP1 as well as transcription factors like p-STAT1, p-STAT3, and p-STAT4. Further bioinformatic analysis indicated that Akt and EGFR are particularly involved in fatty acid metabolism and glycolysis. Indeed, single-cell sequencing analysis confirmed that enrichment of fatty acid and glycolysis metabolism in Lcp1^high^ monocytes/macrophages. Furthermore, Lcp1^high^ cells exhibited enhanced oxidative phosphorylation, chemotaxis, migration, and ATP biosynthesis pathways. *In vitro* experiments confirmed the role of LCP1 in regulating mitochondrial function and fatty acid uptake.

**Conclusions:** These findings contribute to a deeper understanding of LCP1 in the context of ischemic stroke and provide valuable insights into potential therapeutic strategies targeting LCP1 and metabolic pathways, aiming to attenuating neuroinflammation and lymphopenia.

## Introduction

Stroke represents a substantial health burden globally, with more than 795,000 individuals in the United States alone suffering from this condition each year, with approximately 87% of these cases being ischemic stroke 1, 2. The devastating impact of stroke extends beyond mortality, contributing significantly to disability and posing a profound public health challenge 3. As the current treatments such as tissue plasminogen activator (tPA) are effective only within a narrow therapeutic window for a small population 4, there is a critical need for novel, efficient therapeutic strategies, underscoring the urgency to explore novel therapeutic targets.

Ischemic stroke triggers a complex immune response, with neuroinflammation being a key feature that persists for days to weeks post-injury, driven largely by macrophages derived from monocytes and microglia 5-8. The importance of monocyte-derived macrophages (MoDM) and microglia-derived macrophages (MiDMs) has been controversial in brain injury after stroke 9-13, but we and others have demonstrated that MoDMs are crucial in stroke-induced brain injury and functional recovery 14-18. Recruited from peripheral blood monocytes into the ischemic hemisphere, these cells differentiate into macrophages and play contrasting roles in the pathophysiology of ischemic stroke 19-23. Pro-inflammatory macrophages contribute detrimentally to the acute phase, whereas anti-inflammatory macrophages aid in functional recovery in the later stages 14, 24-27. Nevertheless, the associated pathophysiological mechanisms need further clarification.

Stroke not only triggers neuroinflammation, which worsens brain injury, but also leads to immunodepression in peripheral organs like the circulating blood and spleen 28-30. A prominent characteristic of immunodepression is lymphopenia. This immunodepression can persist for weeks to months after stroke, increasing mortality and impeding patient recovery 31, 32. Neuroinflammation exacerbates brain damage, while immunodepression weakens the immune system's ability to combat infections and impairs overall health. Stroke-induced immunodepression heightens the risk of infections and hampers the healing and rehabilitation processes 33, 34. Addressing and understanding this immunodepression or lymphopenia is crucial for improving outcomes and developing interventions to restore immune function after stroke.

Lymphocyte Cytosolic Protein 1 (LCP1), a protein found in immune cells 35, is a promising stroke therapy candidate. It facilitates cell migration, immune responses, and the clearance of pathogens and debris by aiding in actin polymerization, cytoskeletal rearrangements, and phagocytosis 36. Our previous research on monocyte-derived macrophages (MoDMs) regulated by the long non-coding RNA (lncRNA) Maclpil revealed that Maclpil silencing led to LCP1 inhibition, reducing ischemic brain damage and improving stroke outcomes by influencing the migration and phagocytosis capabilities of MoDMs 15. However, the exact role of LCP1 in stroke, especially how it affects stroke outcomes through affecting macrophage functions, remains unexplored.

We hypothesize that LCP1 influences macrophage metabolism, particularly glycolysis and lipid metabolism, and thereby affecting stroke outcomes. Understanding the role of LCP1 in macrophage metabolism during stroke could provide insights into stroke treatment, as previous studies have shown LCP1's potential to influence cellular metabolic processes and immune responses.

In light of these insights, the present study aims to elucidate the role of LCP1 in stroke outcomes, including both brain injury and immunodepression, and the associated pathological mechanisms, specifically focusing on its impact on lipid metabolism within macrophages. Combining *in vitro* and *in vivo* studies with comprehensive bioinformatics analysis using single-cell RNA sequencing data, and the high-dimensional CyTOF technique for protein activation analysis, we aim to unveil the underlying mechanisms of LCP1 modulation of lipid metabolism in relation to stroke. This may shed light on potential therapeutic strategies for ischemic stroke and underscore the potential of LCP1 as a novel therapeutic target.

## Methods and Materials

### Animals

Male C57BL/6J mice at 8-10 weeks of age (22-25 g body weight) were housed at the Department of Laboratory Animal Science, Peking University Medical Center. Five mice were housed in each cage under a 12:12 h light-dark cycle at a temperature of 25 - 27 °C and 40-60% humidity, with freely available food and water. All animal experiments complied with the ARRIVE guidelines and were carried out in accordance with the National Institutes of Health Guide for the Care and Use of Laboratory Animals 15.

### Focal Ischemia Induction and CyTOF Sample Selection in Mice

This study utilized a total of 56 male mice, weighing 22 to 25 g. Focal ischemia was induced using a middle cerebral artery occlusion (MCAo) method, a procedure that was maintained for 45 min, as we previously described 15, 37. To ensure the wellbeing of mice during the surgical procedure, anesthesia was introduced using 5% isoflurane and maintained throughout the surgery with 2% isoflurane. We closely monitored the core body temperatures of the animals via a rectal probe, maintaining it at a consistent 37 °C.

For animals used in the CyTOF detection experiment, we applied specific exclusion criteria to select suitable subjects. The exclusion criteria were as follows: 1) Animals that showed no neurological deficits post-stroke, suggesting insufficient stroke induction. 2) Animals that presented with evidence of surgical subarachnoid hemorrhage, indicating a potential complication in the surgical process. By adhering to these criteria, we ensured a reliable and homogeneous sample pool for our experiments, thereby strengthening the validity and reliability of our subsequent findings.

### Neurobehavioral examination

We utilized the Modified Neurological Severity Score (mNSS) for evaluating the neurobehavioral deficits in our test mice 15. The mNSS is a comprehensive scale ranging from 0, which represents normal neurological function; to 14, indicating the highest degree of neurological deficiency. The scoring includes assessments of motor skills, balance, and sensory reflexes. For assessing motor skills, mice were raised by the tail to observe the movements and torsions of their limbs, which were scored on a scale of 0 to 3. The mice's walking postures were also evaluated, following the same scoring system. The balance of the mice was tested by placing them on a beam and observing if they could maintain their balance, whether their limbs fell off the beam, and whether they could walk on the beam. This was scored on a scale of 0 to 6. Finally, the sensory and reflex tests were conducted by examining pinna and corneal reflexes, scored on a scale of 0 to 2. To ensure impartiality, the neurobehavioral tests were conducted by an investigator who was blinded to the treatment conditions.

### Bioinformatics analysis

The bioinformatics analysis detailed in this section was aimed at comprehensively investigating the gene expression patterns and associated molecular pathways in macrophages following ischemic stroke in mice, with a particular focus on the role of lymphocyte cytosolic protein 1 (LCP1).

For this analysis, we obtained the GSE197731 dataset, obtained from the Gene Expression Omnibus (GEO) 38. We selected cells from the ipsilateral (IL) hemisphere of wild type mice, isolated at 24 h and 48 h post transient middle cerebral artery occlusion (tMCAo) surgery. Single-cell RNA sequencing (scRNA-seq) data were processed using the Seurat package (version 4.1.1) in the R software (version 4.2.1). We filtered the merged datasets from 24 h and 48 h, eliminating cells with fewer than 200 detected genes and those with more than 20% of mitochondrial genes. Subsequent normalization and scaling of the integrated data were performed.

To account for potential batch effects, we utilized Harmony software (version 0.1.0). The processed data were visualized using the Uniform Manifold Approximation and Projection (UMAP) method. We specifically targeted macrophage/monocyte cell subpopulations expressing Lyz2, Ly6c2, and Ccr2, and these cells were further categorized into LCP1^high^ and LCP1^low^ groups based on LCP1 expression levels.

To elucidate functional differences between LCP1^high^ and LCP1^low^ cells, we employed Gene Set Variation Analysis (GSVA) using Hallmark gene set (MSigDB) through the R package GSVA (version 1.44.2) 39, and the correlation between "HALLMARK_FATTY_ACID_METABOLISM" scores and LCP1 expression was analyzed using Pearson's correlation analysis. Further metabolic activity analysis was done using the R package scMetabolism (version 0.2.1) based on mouse KEGG pathways (https://www.genome.jp/kegg/). Gene enrichment analysis was conducted on gene set enrichment analysis (GSEA) software (4.2.3) (http://www.broadinstitute.org/gsea/index.jsp).

The protein-protein interaction (PPI) network was constructed using STRING 40, requiring a minimum interaction score of 0.4, and visualized in Cytoscape v3.9.1 41. To identify significant subnetworks within the PPI, we used the Molecular Complex Detection (MCODE) plug-in of Cytoscape. The applied parameters were: degree cutoff ≥2, node score cutoff: 0.2, K-core: 2, and max depth: 100.

### Culture and knockdown LCP1 in RAW264.7 murine macrophages

In an effort to further our understanding of LCP1 regulation in macrophages, this study utilized the RAW 264.7 murine macrophage cell line 42. Maintaining these cells under controlled conditions, specifically, 37 °C in an environment with 5% CO_2_, we cultured them in Dulbecco's Modified Eagle Medium (DMEM), fortified with 10% Fetal Bovine Serum (FBS) and 1% Penicillin-Streptomycin (P/S). This ensured robust growth and viability within ten passages, as the cells were also confirmed to be mycoplasma-negative.

Our investigation into LCP1 involved targeted gene silencing through RNA interference, facilitated by the transfection of specific siRNA molecules (siRNA ID150197), as per our previous protocol 15. The siRNA, procured from Life Technologies (Thermo Fisher Scientific, Waltham, Massachusetts, USA), was delivered into the RAW 264.7 cells using the HiperFect Transfection Reagent (Qiagen Inc., Germantown, Maryland, USA, Cat^#^ 301704).

### Preparation, polarization and adoptive transfer of bone marrow derived macrophages

The experiment facilitated an exploration of the role of manipulated macrophages within the context of ischemic stroke. Mononuclear cells isolated from bone marrow were cultured at 37 °C in a 5% CO_2_ incubator overnight 17. The cell media was exchanged with M-CSF (10-20 ng/ml) recombinant mouse protein (Thermo Fisher Scientific, Waltham, Massachusetts, USA, Cat^#^ PMC2044) for 6 days. Then the cell media was changed again by adding 1 μg/ml LPS (Sigma‒Aldrich, St. Louis Missouri, USA, Cat ^#^ L2880) to further polarize the macrophages into proinflammatory macrophages, M (LPS). These cells (6 x10^6^ cells) as-prepared above and control macrophages, were collected and adoptively transferred (i.v. injection) into mice after MCAo within 3 h.

### ATP level detection

In order to ascertain the ATP levels within the cells, an Enhanced ATP Assay Kit was utilized (Cat^#^ S0027, Beyotime, China). Following cell lysis with 200 µL of lysis buffer per well in a 6-well plate, the cells were subsequently centrifuged at 12,000 g for 5 min at 4 °C, with the resulting supernatant used for further assays. A standard curve was established using ATP standard solution, appropriately diluted with ATP detection lysate. Initial detections were conducted for concentrations ranging from 0.01 to 10 µM, with adjustments made in subsequent experiments based on the ATP concentration in the sample. The ATP test working fluid was prepared by diluting an appropriate volume of ATP detection reagent with an ATP detection reagent diluent in a 1:4 ratio. Twenty µL of each sample or standard was added to individual wells or tubes. The relative light units (RLU) or counts per minute (CPM) were promptly recorded using a luminometer or liquid scintillation counter with a minimum interval of 2 s between readings. The measurement of ATP levels was critical in assessing the metabolic activity of the cells under investigation.

### CyTOF assay

In this study, a CyTOF assay was employed to perform extensive profiling of cellular phenotypes and functions. The isolation of immune cells from the ischemic hemisphere was achieved through a process previously reported 43, 44, which involved mincing the tissue in RPMI 1640, filtering it through a 70-µm cell strainer, and layering the resultant cell suspension over a Percoll gradient. Post-centrifugation, the interphase cells were collected and prepared for further staining.

Live/dead cell distinction was facilitated by cisplatin staining (Fluidigm Corporation). Following this, individual samples were uniquely barcoded with the Cell-ID 20-Plex Pd Barcoding Kit (Fluidigm) to permit simultaneous analysis of multiple samples. Once barcoded, the cells were exposed to an antibody cocktail to facilitate the detection of cell surface markers and phosphorylated proteins (refer to Table S1 -S2). Notably, all antibodies utilized for CyTOF staining were procured from Fluidigm.

Following staining, the samples were processed using the CyTOF2 system (Fluidigm) located at the Stanford University Shared FACS Facility. The resulting raw data were first calibrated using CyTOF software, and the uniquely barcoded samples were de-multiplexed using Debarcoder software (Fluidigm Corporation, South San Francisco, California, USA). Subsequently, the data were analyzed via Cytobank and SPADE. Cytobank was also employed to generate heatmaps in illustration mode, with the scale range set to global automatic mode, allowing for the comprehensive visualization of the dataset. Through these methods, in-depth cellular profiling was possible, contributing to our understanding of the mechanisms at play in the ischemic microenvironment.

### Flow Cytometric Analysis

Flow cytometry was utilized to determine total lipid content, cellular Reactive Oxygen Species (ROS) level, and fatty acid uptake 45. Staining reagents Bodipy 493, Bodipy C12, and MitoSoX (Cat^#^ D3922, D3822 and M36009, respectively, all purchased from Thermo Fisher) were diluted 1:1000. Following dilution, cells were stained in serum-free culture medium with the staining reagent for 30 min. After the incubation period, cells were washed twice with Phosphate Buffered Saline (PBS) to remove excess reagent. The samples were then analyzed using the Beckman CytoFLEX S flow cytometer (Beckman Coulter, Indianapolis, IN, United States) for quantitative cellular profiling.

### Metabolic flux assays

RAW264.7cells were seeded at 2 × 10^4^ cells/well in normal growth media in a Seahorse XF96 Cell Culture Microplate (Agilent, USA). The plate was then incubated at 37 °C overnight to allow the cells to adhere. The following day, growth media was exchanged with Seahorse Phenol Red-free DMEM and either basal OCR was measured according the manufacturer's instructions. Upon completion of the Seahorse assay, cells were washed three times with pre-warmed phenol-free DMEM media (no FBS) and assay performed using Seahorse XF96 (Agilent, USA).

## Results

### Transferring LCP1-knocking down in MoDMs protects against brain injury induced by stroke

**Figure 1:**
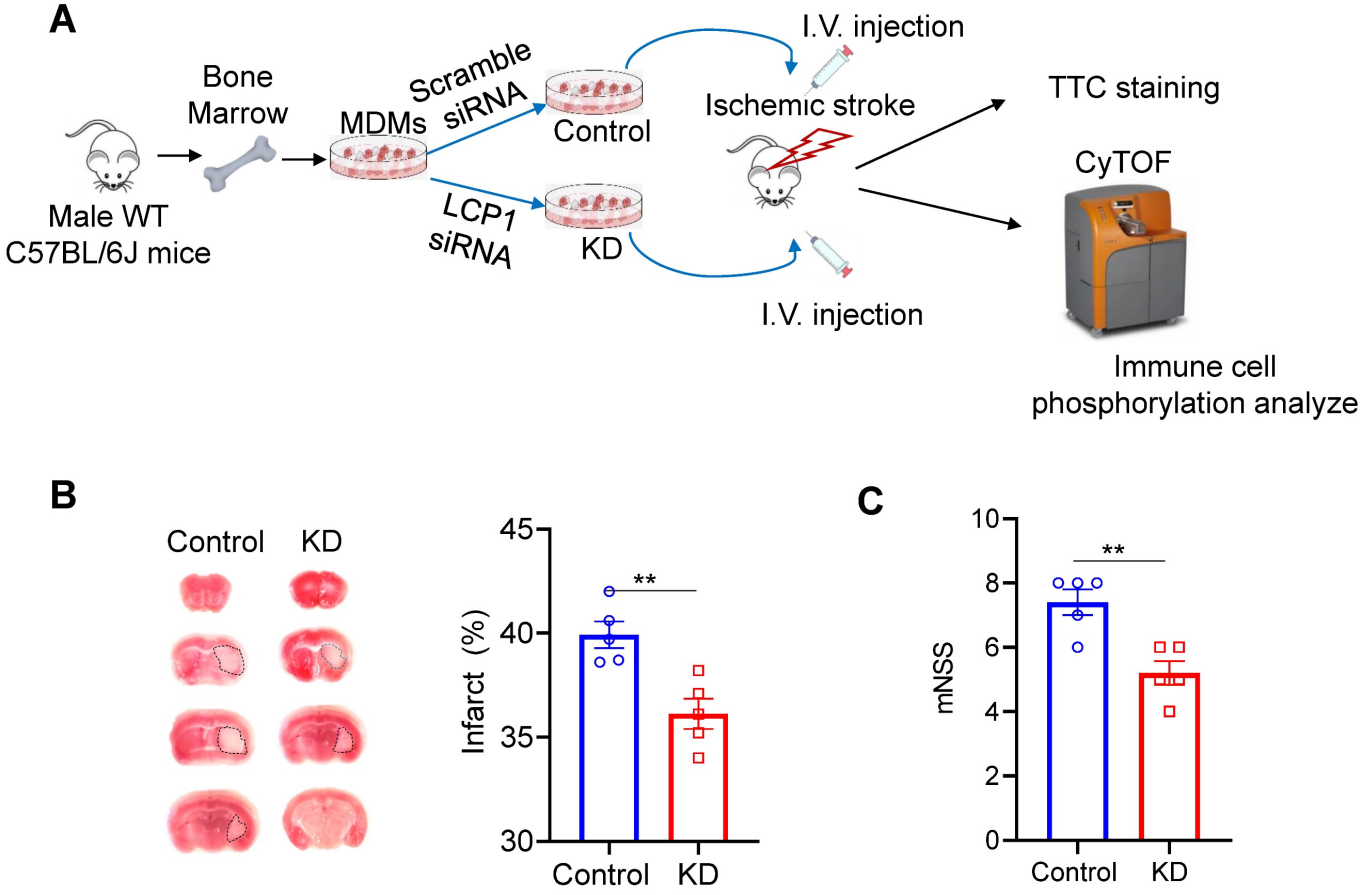
LCP1-silencing macrophages reduces ischemic infarction size and improves neurological behaviors. (A) Overview of the experimental procedures. Primary monocytes were isolated from C57BL/6J mouse bone marrow and polarized into macrophages. The cells were then transfected with either control siRNA or LCP1 siRNA to knock down LCP1 expression. Following middle cerebral artery occlusion (MCAO) surgery, mice were intravenously injected with macrophages transfected with LCP1 siRNA or scramble siRNA. Neurological behaviors, infarction size, and CyTOF analysis were evaluated three days after the MCAo surgery. (B) Representative images of the infarction area identified by TTC staining, along with quantification of the infarction size. (C) Quantification of neurological scores in mice (n=5). Student's t-test was performed, and ** P < 0.01 indicates statistical significance compared to scramble siRNA treatment.

The study utilized primary monocytes sourced from mouse bone marrow, which were polarized into MoDMs and subsequently transfected with either scramble siRNA or LCP1 siRNA (Figure 1A). These modified MoDMs were intravenously introduced into mice post-MCAo surgery. The brain tissues, collected three days post-procedure, were subjected to TTC staining (Figure 1A). The resultant ischemic infarction was significantly less in mice injected with LCP1 siRNA transfected MoDMs (~30% infarction) compared to mice injected with scramble siRNA MoDMs (~40% infarction) (Figure 1B). Neurological scores further corroborated these findings; average scores were at 4.2 in mice treated with LCP1 siRNA MoDMs, contrasting with higher scores in the control group (Figure 1C). In conclusion, adoptive transferring of LCP1-klocking down- in MoDMs demonstrated a notable mitigation of ischemic infarction and neurobehavioral defects.

### LCP1-silenced MoDMs attenuates neuroinflammation in the ischemic brain and lymphopenia associated with immunodepression in the peripheral blood and spleen

In order to delve into the *in vivo* function of LCP1, we collected brain samples three days post-MCAo and proceeded to conduct analyses using the CyTOF technique (Figure 1A). This experiment was designed to incorporate both immune cell surface markers, phosphorylated kinases, and transcription factors to allow a comprehensive identification of immune cell subsets, their intracellular responses, and potential signaling pathways (Table S1). Subsequently, Cytobank was used for data analysis via viSNE and heatmap functions.

**Figure 2:**
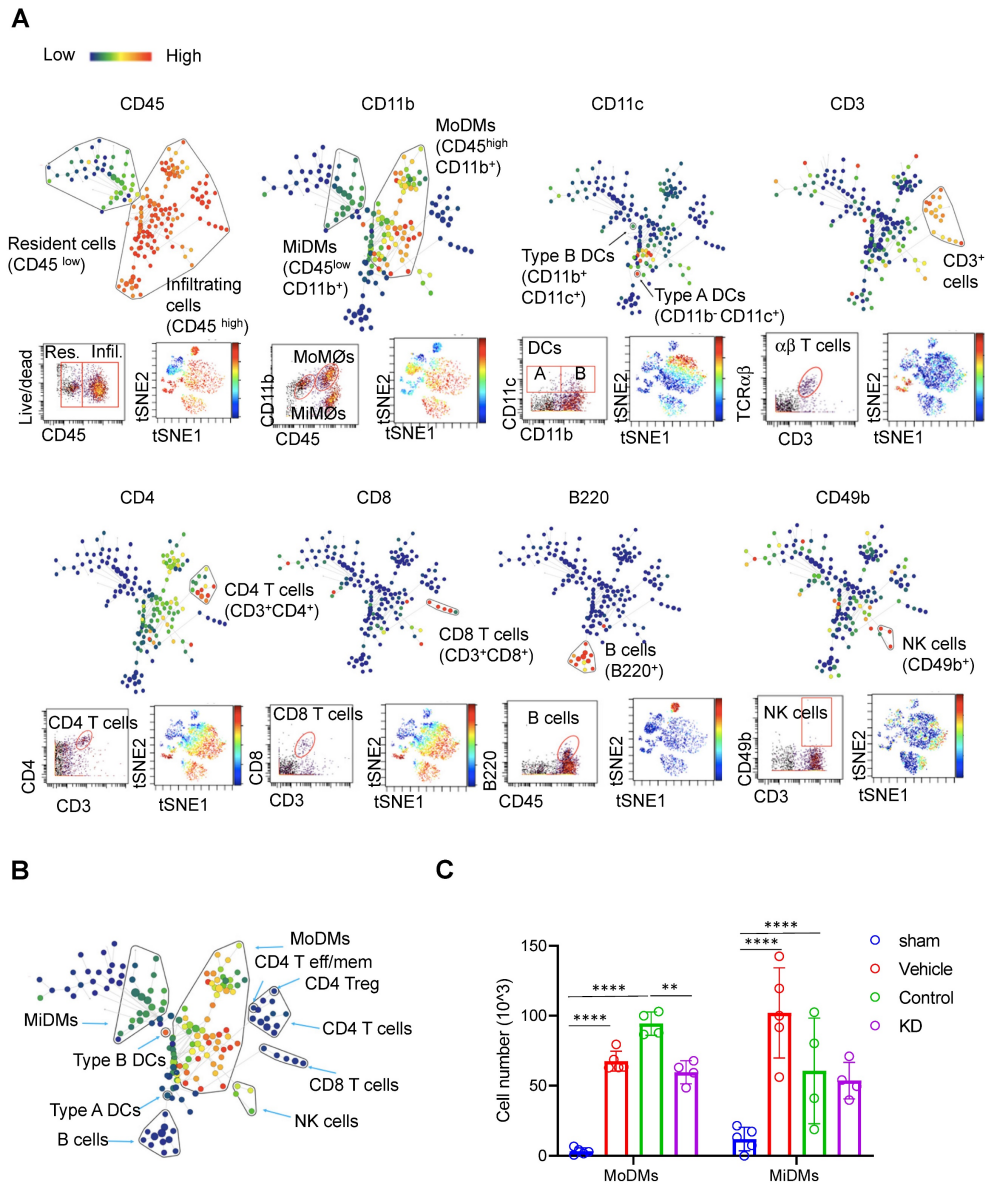
CyTOF analysis reveals immune cell composition and changes in Monocytes Derived Macrophages (MoDMs) and Microglia Derived Macrophages (MiDMs) following ischemic stroke. (A) Representative brain immune cell gating methodology using SPADE (top), dot plots (bottom left), and viSNE (bottom right). In SPADE, the node size reflects the cell numbers, and the color gradient represents the marker expression level. In viSNE, each dot represents a single cell, with color indicating marker intensity. (B) Representative SPADE tree illustrating the identified immune cell types in the brain. (C) Changes in the cell numbers of monocytes derived macrophages (MoDMs) and microglia-derived macrophages (MiDMs) after ischemic stroke. Statistical significance is denoted by ** P < 0.01 and **** P < 0.0001. The "Vehicle group" refers to those receiving only an i.v. injection of PBS, while the "Control group" pertains to those administered an i.v. injection of scramble siRNA.

Through this investigation, we identified 11 immune cell types in the ischemic brain (Figure 2A & Tables S2, S3). Difference in CD45 expression level enabled us to distinguish brain resident from infiltrating immune cells. Resident microglia were denoted by CD45^low^ CD11b^+^, while monocyte-derived macrophages were identified as CD45^high^ CD11b^+^. Various immune cell subsets were identified, including B220^+^ B cells, CD4 T cells, CD8 T cells, CD4 T effector (Tem) cells, CD8 Tem cells, Regulatory T cells (Tregs), NK cells, and dendritic cells (DCs) (Figure 2A - B).

Our results demonstrated that mice receiving LCP1-silenced MoDMs exhibited significantly reduced immune cell numbers within the ischemic hemisphere when compared to mice receiving scramble siRNA treated MoDMs (Figure 2C). A decrease was particularly noticeable in MoDMs, but not in MiDMs in the LCP1 knockdown mice compared with the control group, corroborating our findings that reduction of LCP1 in MoDMs notably attenuates acute experimental ischemic stroke (Figure 2C). In our study, we did evaluate other immune cells, including B cells, T cells, and NK cells. However, their counts were substantially lower in comparison to MoDMs and MiDMs, as depicted in Figure S1. Notably, our observations indicate that LCP1 KD appeared to decrease the numbers of different T cells, DCs, and NK cells, but had no effect on B cell counts.

**Figure 3:**
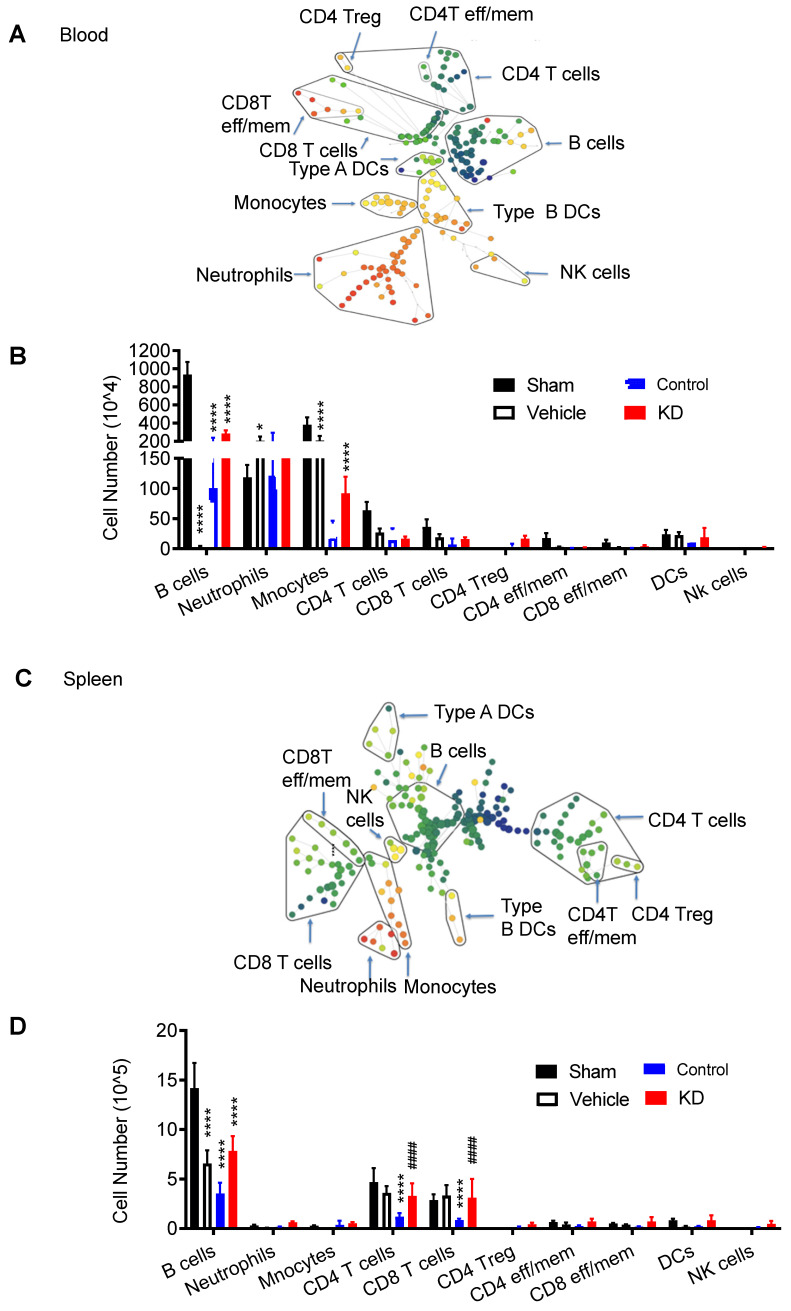
Characterization of immune cell populations in peripheral blood and spleen, 3 day after stroke showing down-regulated LCP1 attenuating peripheral immunodepression induced by ischemic stroke. (A) The representative SPADE tree illustrates the identified immune cell types from peripheral blood. (B) The number changes of various immune cells. (C) The representative SPADE tree illustrates the identified immune cell types from spleen. (D) The number changes of different immune cells. (Vs. to sham group * P < 0.05, *** P < 0.001, **** P < 0.0001. vs. to control group # P < 0.05, #### P < 0.0001).

We also examined the immune response in peripheral organs, including peripheral blood (Figure 3A) and spleen (Figure 3B, Figure S2-S3). CyTOF analysis revealed higher immune cell counts in these peripheral locations in the LCP1 knockdown group compared to the control group, further emphasizing the potential protective role against lymphopenia, which is associated with immunodepression, played by decreased levels of LCP1 in MoDMs.

### LCP1 knockdown promotes the phosphorylation of kinases and transcription factors, which is implicated in glycolysis and fatty acid metabolism

**Figure 4:**
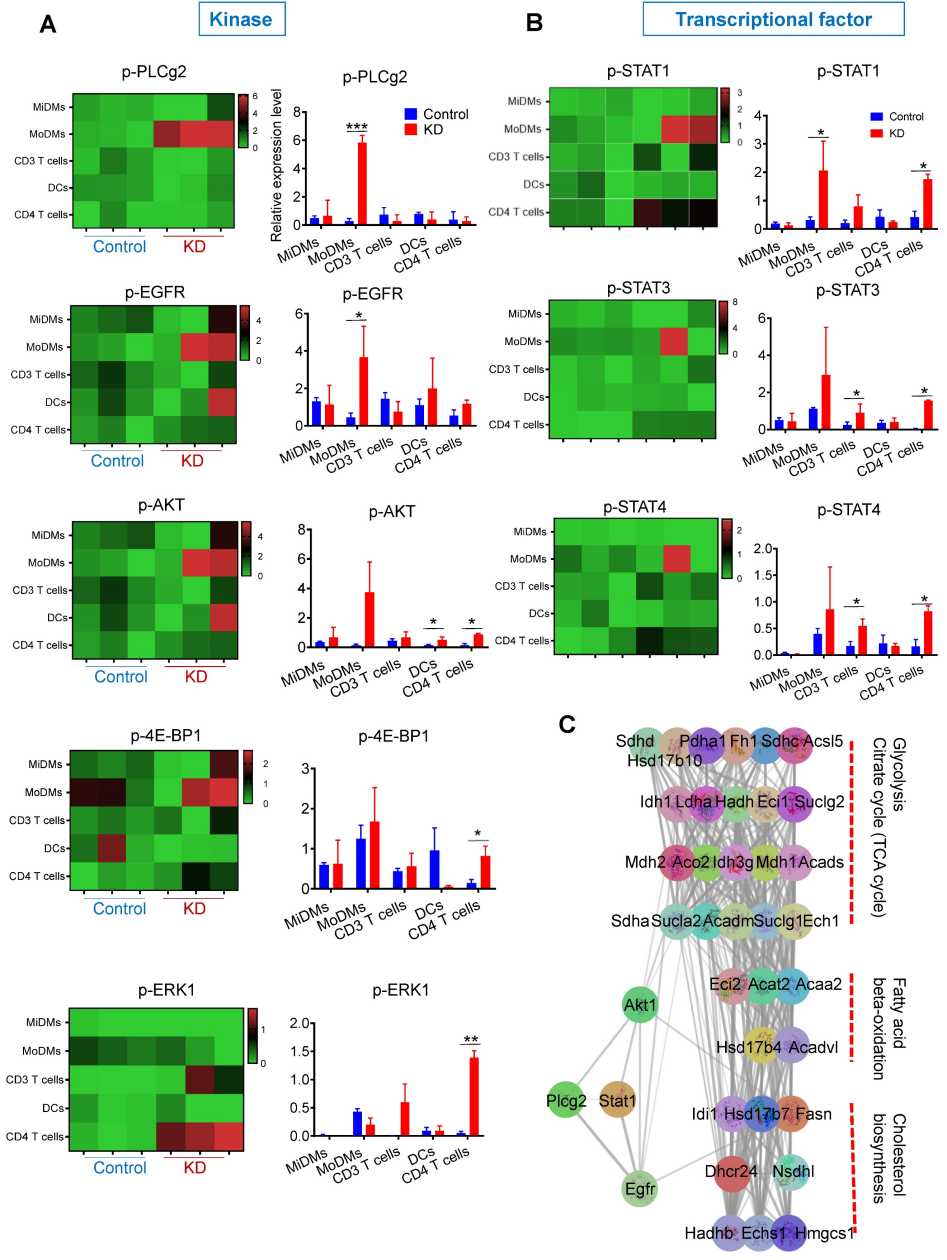
Phosphorylation levels of key kinases in various immune cells and protein-protein interaction network analysis in ischemic brain hemisphere. The figure depicts the phosphorylation levels of major immune cell types in the ischemic brain hemisphere, revealing distinct patterns of kinase activation. Additionally, a protein-protein interaction network analysis highlights key factors involved in lipid metabolism processes, shedding light on the potential molecular mechanisms underlying immune cell responses in ischemic stroke. (A) Phosphorylation levels of key kinases (pPLCg2, pEGFR, pAkt, p4E-BP-1, and pERK1) in immune cell types, including MiDMs, MoDMs, CD3 T cells, DCs, and CD4 T cells. (B) Phosphorylation levels of key kinases (pStat1, pStat3, and pStat4) in immune cell types. (C) Construction of the protein-protein interaction (PPI) network and identification of subnetworks using MCODE. Key factors involved in lipid metabolism processes are grouped into three sets within the subnetworks.

Alongside quantifying immune cell numbers, we investigated the phosphorylation levels of key kinases, including p-PLCg2, p-ERK1/2, p-EGFR, p-AKT, and p4E-BP1 as well as transcription factors like p-STAT1, p-STAT3, and p-STAT4 (Figure 4). Our data showed elevated levels of p-PLCg2 and p-EGFR in MoDMs in mice receiving MDMs with LCP1 knockdown (Figure 4A). Notably, levels of p-AKT, p-4E-BP1, and p-ERK were increased in CD4 T cells (Figure 4A). Expression changes were also observed in transcription factors, predominantly in CD3 T cells and CD4 subsets (Figure 4B). This data suggests that LCP1, as an actin-binding protein, may regulate immune cells via phosphorylation of key kinases, particularly p-PLCg2.

As we previously reported that knocked down lncRNA Maclpil increased the fatty acid metabolism in MoDMs 16, to delve deeper into the potential mechanisms linking key kinases and fatty acid metabolism, we utilized the STRING database to investigate the Protein-Protein Interaction (PPI) network of key kinases and genes involved in fatty acid metabolism enriched in LCP1^high^ macrophages (Figure 4C). Utilizing the Molecular Complex Detection (MCODE) plug-in in Cytoscape, we managed to filter significant modules (Figure 4C). Our analysis revealed a direct correlation between Plcg2 and two key elements, Akt1 and EGFR. These are connected with genes implicated in fatty acid metabolism, such as fatty acid synthase (Fasn). Moreover, Akt1 and EGFR share associations with genes involved in glycolysis, potentially providing intermediate metabolites to support fatty acid metabolism. These findings hint at a possible role of LCP1 in regulating immune cells through these kinases within fatty acid metabolism.

### Glycolysis and fatty acid metabolism are highly enriched in Lcp1^high^ monocytes/ macrophages in the ischemic brain hemisphere

**Figure 5:**
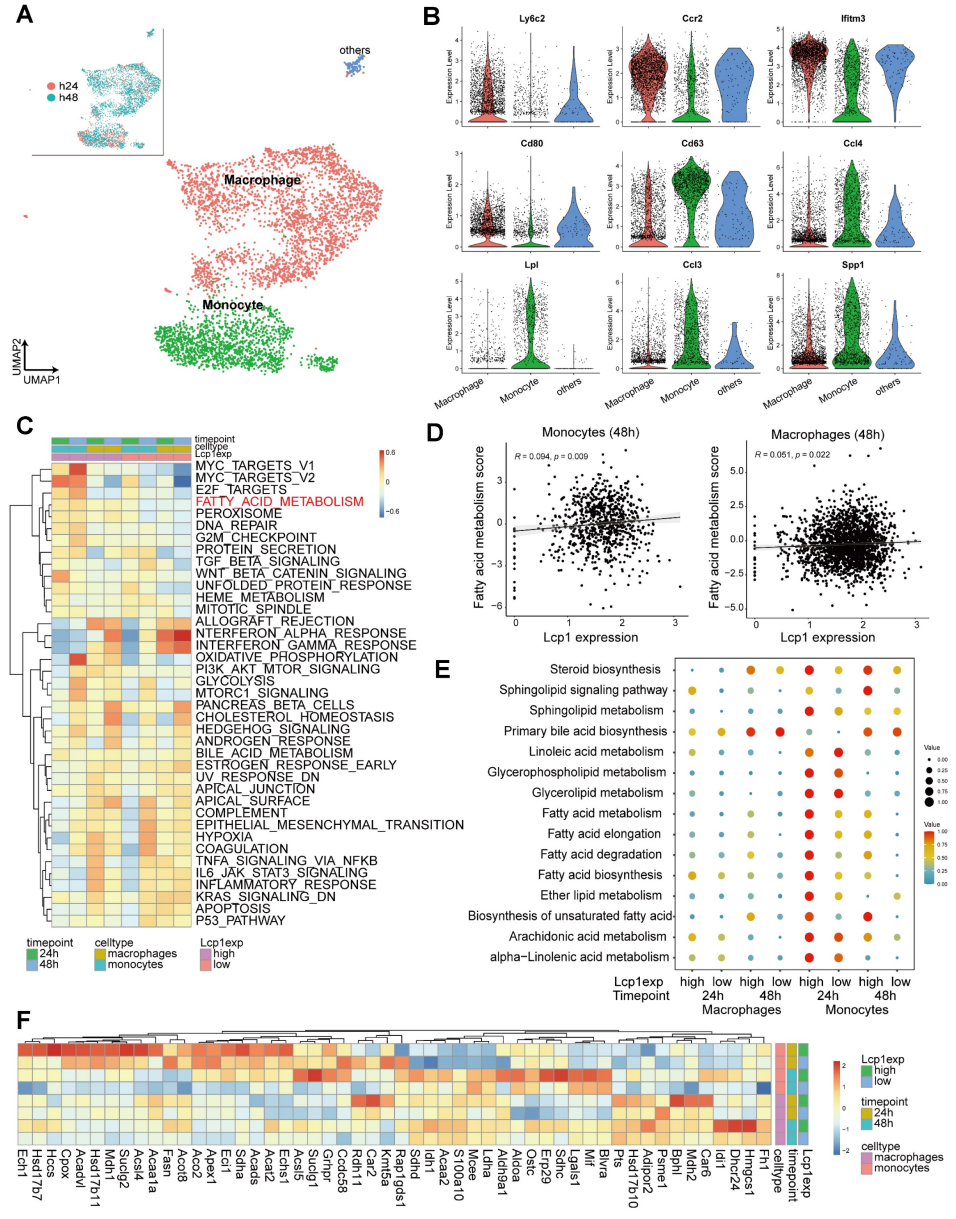
Single cell RNA-seq reveals the metabolic characteristics of LCP1^high^ macrophages and monocytes in the stroke brain. (A) Uniform Manifold Approximation and Projection (UMAP) plot showing clusters of macrophages and monocytes at 24 h and 48 h after MCAo. (B) Violin plots showing representative markers for macrophages and monocytes. (C) Heatmap showing scores of top 10 hallmark gene sets enriched in LCP1^high^ and LCP1^low^ macrophages and monocytes at 24 h and 48 h after MCAo calculated by gene set variation analysis (GSVA). (D) Pearson correlation of fatty acid metabolism score and LCP1 expression in monocytes/macrophages at 48 h after MACo. Monocytes (left), and macrophages (right) at 48 h after stroke. (E) Dot plots showing the expression scores of lipid metabolism related pathways in LCP1^high^ and LCP1^low^ macrophages and monocytes at 24 h and 48 h after MCAo. (F) Heatmap showing the expression of fatty-acid-metabolism-related genes.

Aiming to delineate the metabolic landscape of monocytes/macrophages expressing high levels of LCP1 within the stroke-affected hemisphere, we initiated our research with a bioinformatics analysis using the publicly available single-cell dataset (GSE197731) 38. We honed in on data from the isolated ipsilateral (IL) hemisphere cells of mice, collected at 24 h and 48 h subsequent to the transient MCAo surgery (Figure S4A - B). By focusing on the expression of Cd68, Cx3cr1, Ly6c, Tgfb1, Lyz2, and Ccr2, we were able to pinpoint clusters that represented monocytes or macrophages (Figure S4C). Subsequently, these monocytes and macrophages were separately identified and clustered (Figure 5A), in alignment with the expression of marker genes such as Ly6C2, CCr2, ifitm3, Cd80, Cd63, Ccl4, Lpl, Ccl3, and Spp1 (Figure 5B).

We further categorized these cells into LCP1^high^ and LCP1^low^ populations, based on LCP1 expression levels at 24 h and 48 h post-ischemia (Figure S4D). Intriguingly, our GSVA revealed a distinct enrichment of fatty acid metabolism in LCP1^high^ monocytes/macrophages as compared to LCP1^low^ cell populations, at both time points (Figure 5C). Drawing on this single-cell sequencing data, we demonstrated a positive correlation between LCP1 expression and fatty acid metabolism in both monocytes and macrophages (Figure 5D).

Furthermore, we evaluated the lipid metabolism-related pathways in these cell types using scMetabolism 46, finding an apparent upregulation in both LCP1^high^ monocytes and macrophages. Particularly notable was the stark disparity observed 24 h after ischemia between LCP1^high^ and LCP1^low^ monocytes in terms of fatty acid metabolism, biosynthesis, elongation, and degradation (Figure 5E, Figure S4E). These observations align with prior reports which suggested that LCP1 deficiency amplified lipid catabolism 47, reinforcing the reliability of our single-cell sequencing analysis data. The heatmap provided further clarity by elucidating key gene expression patterns (Figure 5F). For instance, the expression of enoyl-coenzyme A hydratase 1 (ECH1), a gene implicated in mitochondrial fatty acid β-oxidation 48, was significantly elevated in LCP1^high^ monocyte populations 24 h post-ischemia. In LCP1^high^ macrophages, three-hydroxy-3-methylglutaryl coenzyme a synthase (HMGCS) levels were markedly increased 48 h after ischemia (Figure 5F). Collectively, these findings indicate an enrichment of fatty acid metabolism in both monocytes and macrophages expressing high levels of LCP1.

### LCP1^high^ is enriched in cellular oxidative phosphorylation and migration pathways in monocytes, but it is enriched in oxidative phosphorylation and ATP biosynthesis in macrophages

**Figure 6:**
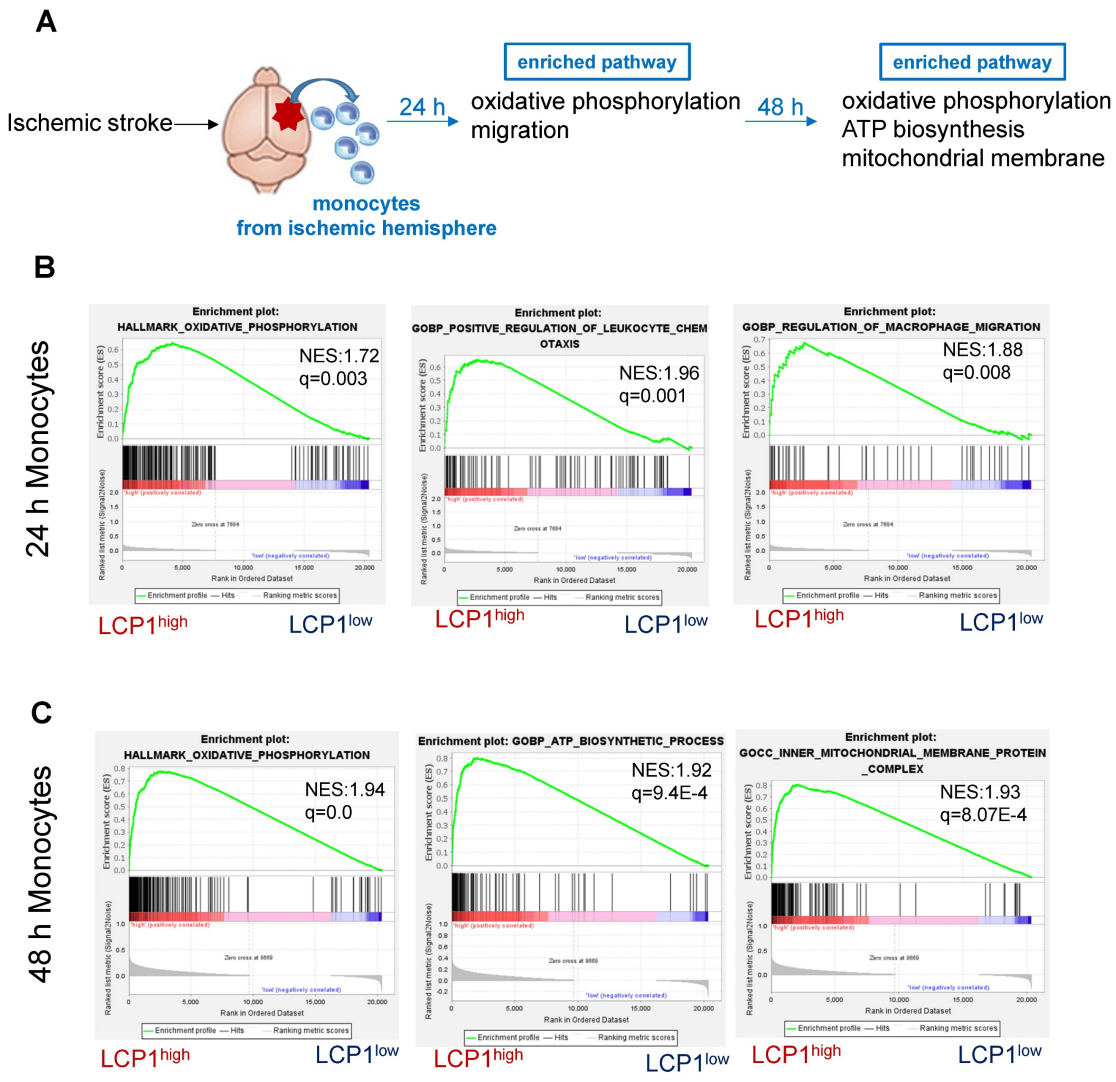
Gene set enrichment analysis (GSEA) of pathways in LCP1^high^ and LCP1^low^ monocytes after MCAo. (A) The diagram indicates the enriched pathways in LCP1^high^ monocytes in damaged brain hemisphere, using GSEA of biological processes at 24 h and 48 h after ischemia. (B) GSEA of biological processes between LCP1^high^ and LCP1^low^ monocytes at 24 h, the oxidative phosphorylation, and cell migration processes are highly enriched in LCP1^high^ monocytes. (C) GSEA of biological processes between LCP1^high^ and LCP1^low^ monocytes at 48 h. Oxidative phosphorylation, ATP biosynthetic process and mitochondrial membranes protein process are highly enriched in LCP1^high^ monocytes.

We next applied the GSEA analysis to determine the pathways associated with LCP1 expression at 24 h and 48 h, two time points (Figure 6A). Twenty-four hours after ischemic damage, the oxidative phosphorylation pathway was highly enriched in LCP1^high^ monocytes, suggesting that mitochondria were required to produce more energy when LCP1 is highly expressed (Figure 6B). In addition, the leukocyte chemotaxis and migration pathways were both enriched in LCP1^high^ monocytes (Figure 6B), indicating that monocytes highly expressing LCP1 not only had the capacity to produce chemokines to attract other immune cells, but also had better migration abilities into the damaged area. These data were supported by our previous finding that knockdown of LCP1 significantly decreased the migration capabilities of bone marrow-derived macrophages 15, 49. Forty-eight hours after the damage, the oxidative phosphorylation, ATP biosynthesis and mitochondrial membrane pathways were highly enriched in LCP1^high^ monocytes (Figure 6C).

**Figure 7:**
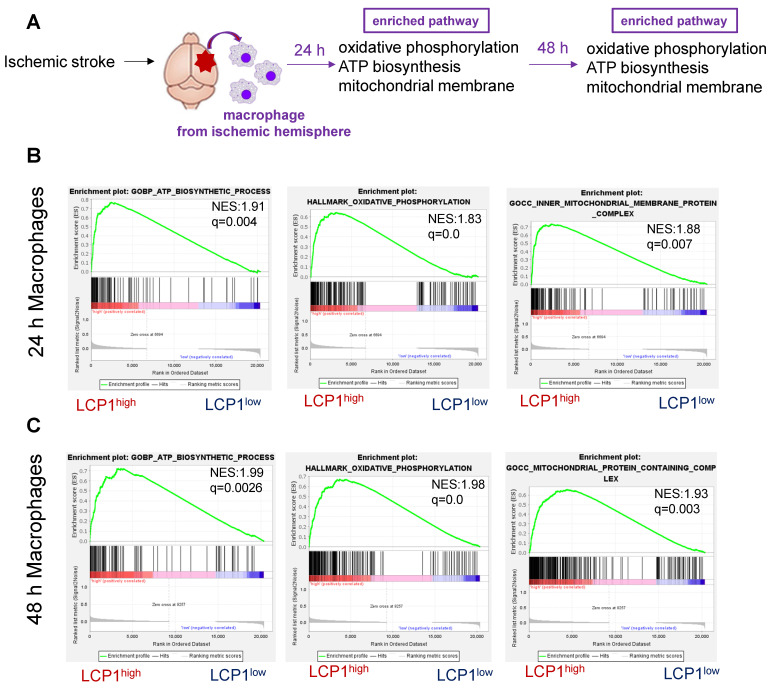
Gene set enrichment analysis (GSEA) of pathways in LCP1^high^ and LCP1^low^ macrophages after MCAo. (A) The diagram indicates the enriched pathways in LCP1^high^ macrophages in damaged brain hemisphere, using GSEA of biological processes at 24 h and 48 h after ischemia. (B) GSEA of biological processes between LCP1^high^ and LCP1^low^ macrophages at 24 h, the oxidative phosphorylation, ATP biosynthesis and mitochondrial processes are highly enriched in LCP1^high^ macrophages. (C) GSEA of biological processes between LCP1^high^ and LCP1^low^ macrophages at 48 h. Oxidative phosphorylation, ATP biosynthetic process and mitochondrial membranes protein process are highly enriched in LCP1^high^ macrophages.

Using a similar strategy, we also analyzed the LCP1^high^ macrophage populations in ischemic brain at 24 h and 48 h after ischemia (Figure 7A). Our bioinformatics analysis further showed that in the ischemic hemisphere, oxidative phosphorylation, ATP biosynthesis and mitochondrial membrane pathways were highly enriched in LCP1^high^ macrophages 24 h (Figure 7B) and 48 h (Figure 7C) after ischemic damage.

### Knockdown of LCP1 negatively affects macrophage mitochondrial function that are responsible for oxidative phosphorylation and ATP biosynthesis

**Figure 8:**
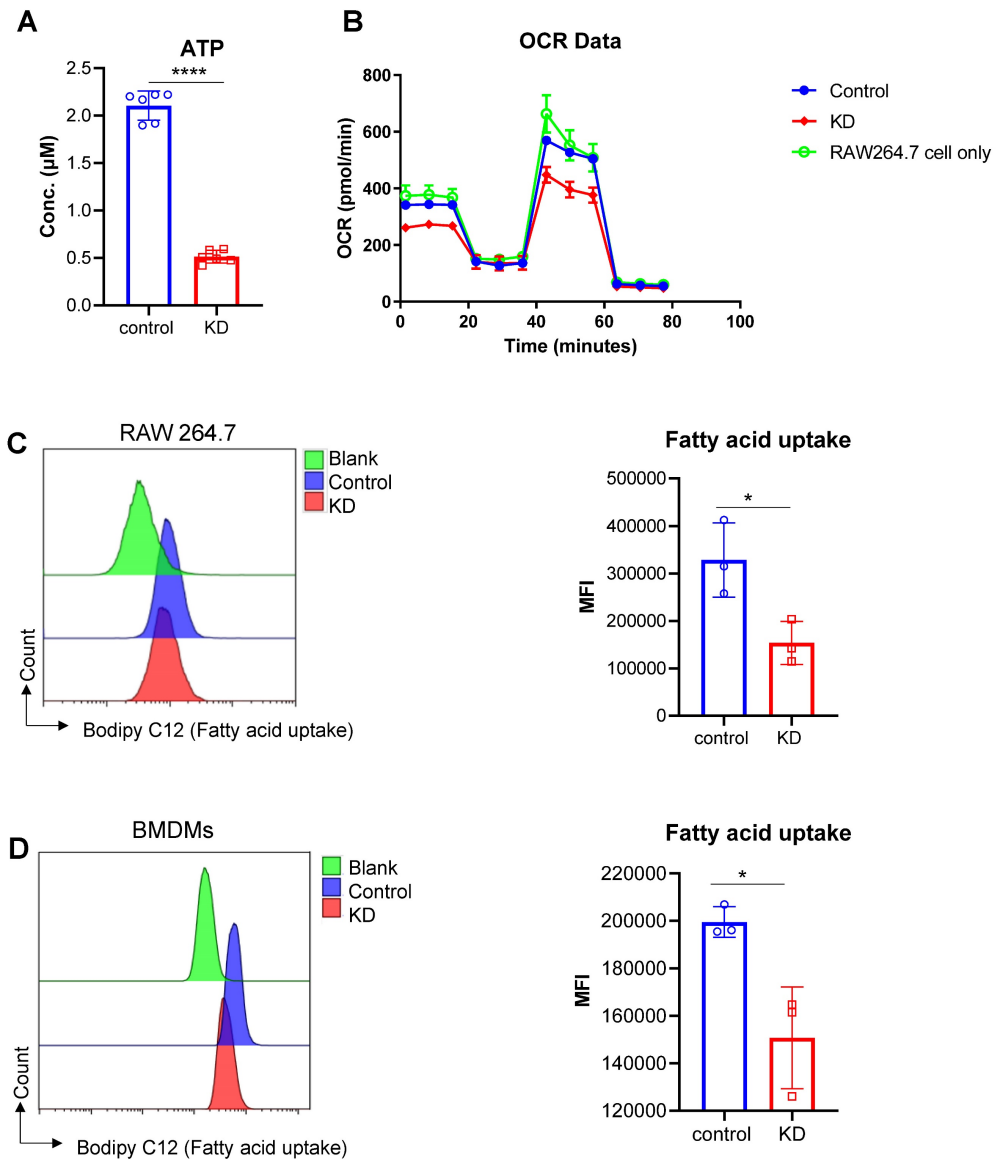
Effects of LCP1 Knockdown on Cellular Metabolism. (A) ATP levels in the control group (control) are significantly higher than the level in LCP1 knockdown group (siRNA). (B) OCR data post mitochondrial stress test using RAW264.7 cell lines, the green line stands for RAW264.7 cells without transfection; the blue line stands for RAW264.7 cells transfected with scramble siRNA; and red line stands for RAW264.7 cells transfected with LCP1 siRNA. (C) Representative flow cytometric overlay histograms of Bodipy C12 staining in RAW264.7 cells (left). Quantification of Bodipy C12 expression levels, representing fatty acid uptake, is shown in the right panel. (D) Representative flow cytometric overlay histograms of Bodipy C12 staining using primary BMDMs (left). Quantification of Bodipy C12 expression levels (right). Student's t-test was used for statistical analysis, with * P < 0.05 and **** P < 0.0001 compared to scramble siRNA treatment.

Mitochondrial dysfunction is an important pathological process in the setting of ischemic brain injury. Bioinformatics analysis indicated that LCP1^high^ cell populations were closely associated with ATP synthesis, oxidative phosphorylation and mitochondrial membrane proteins. To further test this bioinformatics result, we used RAW264.7 cells to evaluate the ATP level in the LCP1 knockdown group and scramble siRNA control group. Our data showed that when LCP1 expression was downregulated, the level of ATP was also decreased, suggesting that mitochondrial functions, especially ATP synthesis, were blocked (Figure 8A). To confirm this metabolic character, the classical metabolic assay Seahorse O2 consumption rate (OCR) was performed using RAW264.7 cells. While there was no significant difference between RAW264.7 cells and scramble siRNA control group, the data showed that when LCP1 was knocked down (KD) the O2 consumption rate (OCR) was significantly lower than control (Figure 8B). We next evaluated the cell capabilities to uptake fatty acid in LCP1 knockdown cells, the data showed that the cell capabilities to uptake fatty acid were also down-regulated when LCP1 was knocked down (Figure 8C). This data was also confirmed in primary BMDMs (Figure 8D).

## Discussion

In this investigation, the protective role of LCP1 knockdown in MoDMs against ischemic brain injury, neuroinflammation, and immunodepression was clearly established. Key findings illustrated that LCP1 knockdown in MoDMs not only reduced ischemic infarction and neurobehavioral defects, but also led to a marked decrease in the number of immune cells in the ischemic hemisphere. In addition, it also attenuated lymphopenia, indicating it may inhibit stroke induced immunodepression. Moreover, the underlying mechanisms associated with LCP1 knockdown were explored, revealing alterations in the phosphorylation levels of critical kinases and transcription factors, correlating with genes involved in fatty acid metabolism. Single-cell sequencing data underlined the enrichment of fatty acid metabolism in LCP1^high^ monocytes/macrophages, alongside an enrichment of oxidative phosphorylation, migration pathways in monocytes, and oxidative phosphorylation and ATP biosynthesis pathways in macrophages. The effect of LCP1 on metabolic regulation was further corroborated by *in vitro* experiments, underscoring the potential therapeutic implications of these discoveries in the context of ischemic stroke.

In this research, we further clarified the role of LCP1, building upon existing literature and diversifying our understanding of its influence on MoDMs 16. Preceding studies established LCP1's involvement primarily in cell movement and structure 35, 50, 51, aligning with our observations of its role in immune cell migration. Yet, we embarked on a novel exploration of LCP1's functions, unveiling its role in metabolic regulation and the notable impact on ischemic brain injury, areas less explored in prior work.

The significantly altered immune cell behavior witnessed through LCP1 knockdown illuminated its influence on signaling and metabolism 36, 50. Notably, LCP1 knockdown prompted changes in the phosphorylation patterns of critical signaling kinases and transcription factors 52-54 within MoDMs. The genes affected by these changes had strong ties to fatty acid metabolism and glycolysis. This novel finding implicates LCP1 in the intricate interplay of immunology and metabolism, potentially uncovering new targets for therapeutic interventions.

Additionally, our study dissects the role of high LCP1 expression, known as LCP1^high^, in metabolic and migration pathways. The single-cell sequencing data highlighted an enrichment of oxidative phosphorylation and ATP biosynthesis pathways in LCP1^high^ monocytes and macrophages. This not only underscores LCP1's critical role in cellular energy production but also suggests a possible link between LCP1 expression and the efficiency of these metabolic pathways 55, 56. Concurrently, we observed an enrichment of migration-related genes in LCP1^high^ monocytes, correlating LCP1 expression levels with immune cell migration.

Collectively, these findings suggest that LCP1's involvement in these vital pathways may contribute to its observed influence on neuroinflammation and ischemic brain injury. However, further exploration is necessary to fully comprehend these intricate connections and their broader implications for disease therapy and management.

The implications of our findings are profound, expanding the current understanding of LCP1's role in ischemic stroke and potentially informing the development of novel therapeutic strategies.

First, our study uncovers a new facet of LCP1's role in ischemic stroke, beyond its well-established function in cell movement and structure 51. Specifically, we revealed LCP1's significance in metabolic regulation within MoDMs and the consequent impact on ischemic brain injury and neuroinflammation. The connection between LCP1 expression levels and the severity of ischemic outcomes, demonstrated through LCP1 knockdown models, reinforces the potential of LCP1 as a viable therapeutic target. Furthermore, our findings link LCP1 expression to the enrichment of oxidative phosphorylation, ATP biosynthesis, and migration pathways, which may further guide research in dissecting the molecular mechanisms underlying ischemic stroke. Thus, the study refines our understanding of LCP1's intricate roles in ischemic stroke and sets the stage for future investigations.

Second, the nuanced understanding of LCP1 garnered from our research could prove crucial in informing therapeutic strategies for stroke. By unveiling the significance of LCP1 in metabolic regulation and immune cell migration, this study identifies potential intervention points that could be manipulated to mitigate the deleterious effects of stroke 57. Furthermore, the observation that LCP1 knockdown leads to decreased immune cell infiltration and less severe ischemic outcomes suggests that strategies aimed at modulating LCP1 expression or function may have therapeutic potential. Moreover, our finding that LCP1^high^ cells are particularly enriched in crucial metabolic pathways raises the intriguing possibility that interventions could be designed to target these pathways, thereby influencing LCP1's impact on ischemic injury. While the path to clinical application is long and requires further exploration and validation, our findings present a promising direction for the development of novel therapeutic strategies to combat ischemic stroke.

The clinical significance of attenuating immunodepression in stroke patients is profound 31, 58. Post-stroke immunodepression increases susceptibility to infections, impairs healing and recovery processes, and negatively impacts patient outcomes 59, 60. By targeting LCP1 and its associated metabolic pathways, it may be possible to restore immune cell function, improve immune responses, and mitigate the risk of infections in stroke patients. This could lead to better recovery, reduced morbidity, and improved overall clinical outcomes. Nevertheless, the mechanisms underlying the attenuation of immunodepression by LCP1 knockdown in monocyte-derived macrophages (MoDMs) are not understood. It is likely that the protective effects of LCP1 knockdown against stroke-induced brain injury contribute to the reduction in immunodepression. By decreasing ischemic infarction and improving neurological outcomes, LCP1 knockdown indirectly helps preserve immune cell numbers, including T cells and B cells. Additionally, LCP1 may interact with other cell types involved in the immune response, influencing immune cell homeostasis. Further research is needed to fully elucidate these mechanisms and explore the clinical significance of LCP1 knockdown in the context of immunodepression.

Although our study offers valuable insights, it does contain inherent limitations. The knockdown technique we used may not fully emulate intricate physiological gene regulation, and extrapolation from *in vitro* to *in vivo* settings should be undertaken with caution 61. Further, uncertainties persist, including the precise mechanisms of LCP1's role in metabolic and migratory pathways and how these findings may apply to different cell types, experimental stroke models, or human patients. These considerations underscore the need for future research to refine our understanding of LCP1's role in ischemic stroke.

While our study has advanced the understanding of LCP1's role in ischemic stroke, it also raises intriguing questions that warrant future exploration. A more detailed characterization of the interplay between LCP1 and metabolic pathways in immune cells could help elucidate the precise mechanisms through which LCP1 affects stroke outcomes. There's also a need for future research to investigate how LCP1 affects other cell types and their involvement in ischemic brain injury. Moreover, our findings could be further verified and extended through the use of different experimental stroke models 62, including *in vivo* models and human patient samples. Importantly, translating these findings into a therapeutic context will require assessing the safety and efficacy of LCP1-targeted interventions, which could potentially open a new avenue for ischemic stroke treatment. Through these avenues, we anticipate that further studies can continue to build upon our work and provide a more comprehensive understanding of LCP1's role in ischemic stroke.

In summary, our study provides important insights into the role of LCP1 in ischemic stroke and its potential implications for therapeutic interventions. We have demonstrated the significant protective effect of LCP1 knockdown in monocyte-derived macrophages, reducing ischemic infarction and neurobehavioral defects. It inhibits neuroinflammation and attenuates immunodepression. Our findings also highlight the influence of LCP1 on immune cell signaling and metabolism, with potential implications for immunomodulation and metabolic reprogramming in ischemic stroke. Moreover, the enrichment of oxidative phosphorylation, chemotaxis, migration, and ATP biosynthesis pathways in LCP1^high^ cells further underscores the importance of LCP1 in cellular energy production and immune cell behavior. Moving forward, future research should focus on elucidating the precise molecular mechanisms underlying the role of LCP1 in metabolic regulation and immune cell function. Additionally, the translation of these findings into therapeutic strategies for ischemic stroke requires further investigation and assessment of the safety and efficacy of LCP1-targeted interventions. By addressing these questions, future studies can continue to advance our understanding of LCP1's involvement in ischemic stroke and potentially pave the way for novel therapeutic approaches.

## Supplementary Material

Supplementary figures and tables.

## Abbreviations

LCP1: lymphocyte cytosolic protein 1
LncRNA: long non-coding RNA
MoDMs: monocyte-derived macrophages
tPA: tissue plasminogen activator
MiDMs: microglia-derived macrophages
MCAo: middle cerebral artery occlusion
mNSS: modified neurological severity score
IL: ipsilateral
GEO: gene expression omnibus
scRNA-seq: single-cell RNA sequencing
UMAP: uniform manifold approximation and projection
GSVA: gene set variation analysis
PPI: protein-protein interaction
MCODE: molecular complex detection
FBS: fetal bovine serum
P/S: penicillin-streptomycin
ECH1: enoyl-coenzyme A hydratase 1
HMGCS: hydroxy-3-methylglutaryl coenzyme a synthase
ROS: reactive oxygen species
Tem cells: CD4 T effector
Tregs: Regulatory T cells
DCs: dendritic cells
