# Supplementary material for: LCP1 knockdown in monocyte-derived macrophages: mitigating ischemic brain injury and shaping immune cell signaling and metabolism
Source: Theranostics. 2024 Jan 1;14(1):159–75. doi: 10.7150/thno.88678 (PMC10750214; doi:10.7150/thno.88678)
Supplement: Supplementary file 1 — Supplementary figures and tables. [file thnov14p0159s1.pdf]

SUPPLEMENTAL DATA

Table S1 CyTOF antibody Panel

**Cell identify markers**		
**Antibodies**	**Clone**	**Metal lab**
Anti-mouse Ly6G	IA8	141Pr
Anti-mouse CD11c	N418	142Nd
Anti-mouse Ly6C	H1.4	162Dy
Anti-mouse CD45	30-F11	147Sm
Anti-mouse CD11b	M1/70	148Nd
Anti-mouse B220	RA3-6B2	176Yb
Anti-mouse CD25	3C7	151Eu
Anti-mouse CD3	145-2C11	165Ho
Anti-mouse F4/80	BM8	159Tb
Anti-mouse CD45RB	C363.16A	145Nd
Anti-mouse CD8	53-6.7	168Er
Anti-mouse TCRβ	H57-597	169Tm
Anti-mouse CD49b	HMa2	170Er
Anti-mouse CD44	IM7	150Nd
Anti-mouse CD4	RM4-5	172Yb
DNA staining		191/193Ir
Live/dead sating		195Pt
Barcoding		102-110Pd
Phosphorylation markers		
Anti-mouse p4E-BP1	236B4	149Sm
Anti-mouse pAKT	D9E	152Sm
Anti-mouse pS6	S235/S236	175Lu
Anti-mouse pPLCg2	K86-889.37	144Nd
Anti-mouse pEGFR	D7A5	146Nd
Anti-mouse pStat1	4a	153Eu

Anti-mouse pStat3	4	158Gd
Anti-mouse pStat4	38	174Yb
Anti-mouse p38	T180/Y182	156Gd
Anti-mouse pERK1/2	D13.14.4E	171Yb

Table S2 Identified immune cells

**Cell Types**	**Defined Markers**
Monocyte derived macrophages (MoDMs)	$CD45^{hi}CD11b^{+}$
Microglia derived macrophages (MiDMs)	$CD45^{lo}CD11b^{+}$
DCs	$CD45^{+}Cd11C^{+}$
Monocytes	$CD45^{+}Ly6G^{-}$ $Ly6C^{+}$
B cells	$CD45^{+}B220^{+}$
CD8 T cells	$CD45^{+}$ $CD3^{+}CD8^{+}$
CD4 T cells	$CD45^{+}$ $CD3^{+}CD4^{+}$
Neutrophils	$CD45^{+}Ly6G^{+}$ $Ly6C^{-}$
CD4 T_{EM}	$CD3^{+}CD4^{+}CD44^{+}$
CD8 T_{EM}	$CD3^{+}CD8^{+}CD44^{+}$
NK cells	$CD45^{+}$ $CD49^{+}$

Table S3 The changed phosphorylation status of immune cells in ischemic hemisphere

Phosphorylation Markers	Immune cell type have altered expression level
pEGFR	MoDMs
p4E-BP1	CD4 T cells
pStat4	CD3 T cells , CD4 T cells
pStat1	MoDMs
pERK1	CD4 T cells
pS6	CD3 T cells , CD4 T cells
pStat3	CD3 T cells , CD4 T cells
pPLcg2	MoDMs
pAKT	CD4 T cells, MoDMs, DCs
pP38	CD3 T cells , CD4 T cells

Supplemental Figure

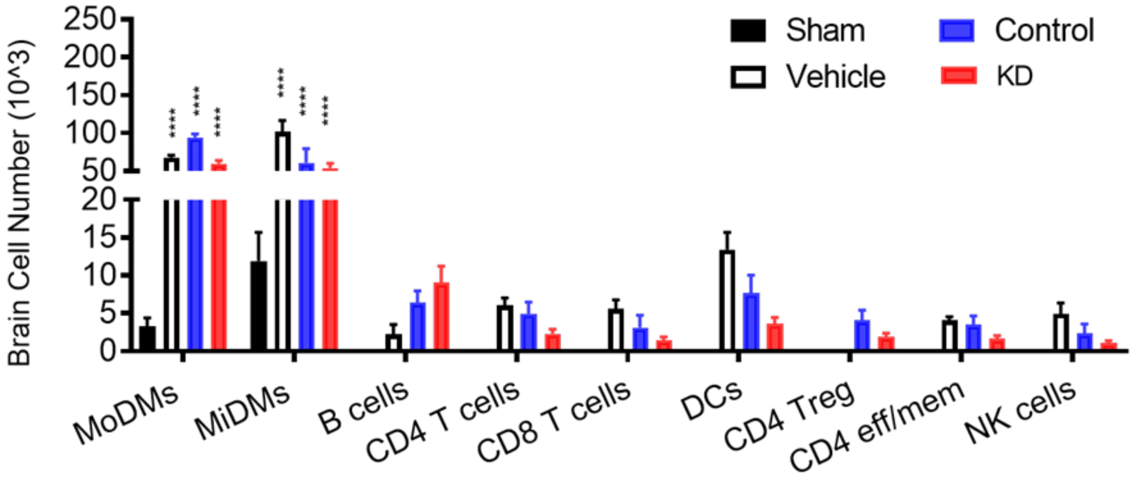

Figure S1. Characterization of post-stroke immune cell populations. Variations in the numbers of distinct immune cells in the ischemic brain hemisphere were noted (compared to the sham group, ***P<0.001).

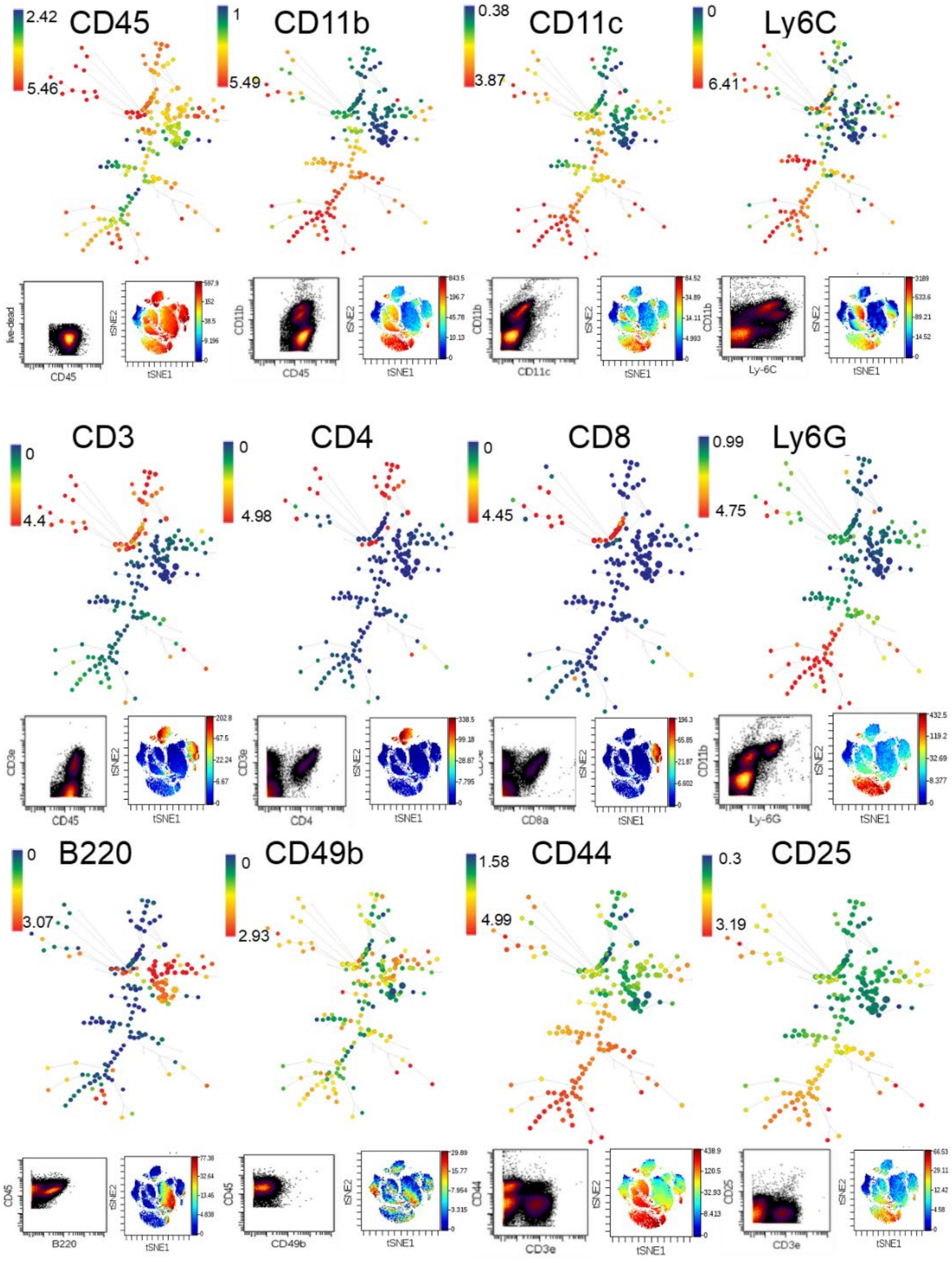

Figure S2. Analysis of blood immune cells using mass cytometry: SPADE representation (top), event plots (bottom left), and viSNE visualization (bottom right).

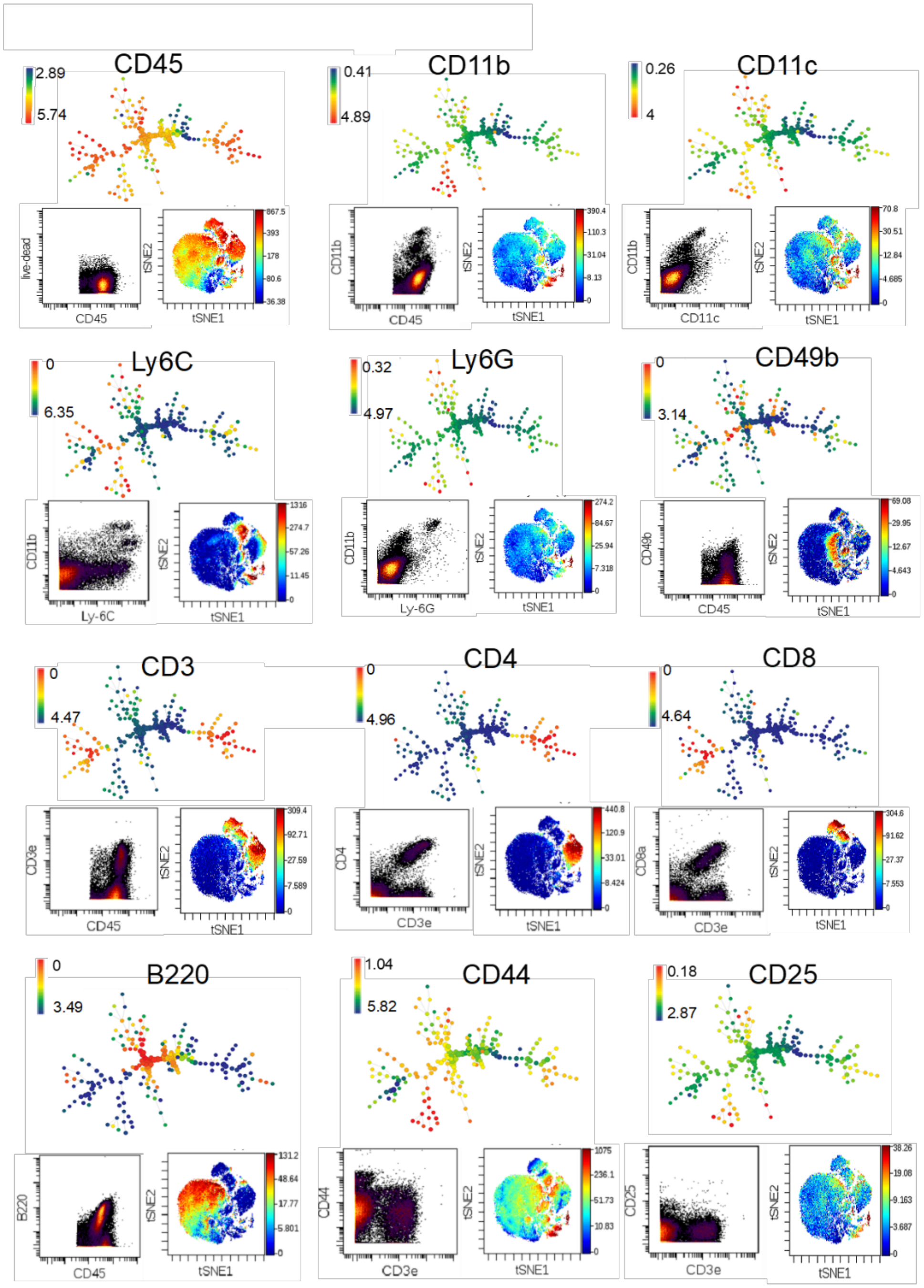

Figure S3. Analysis of spleen immune cells via mass cytometry: SPADE representation (top), event plots (bottom left), and viSNE visualization (bottom right).

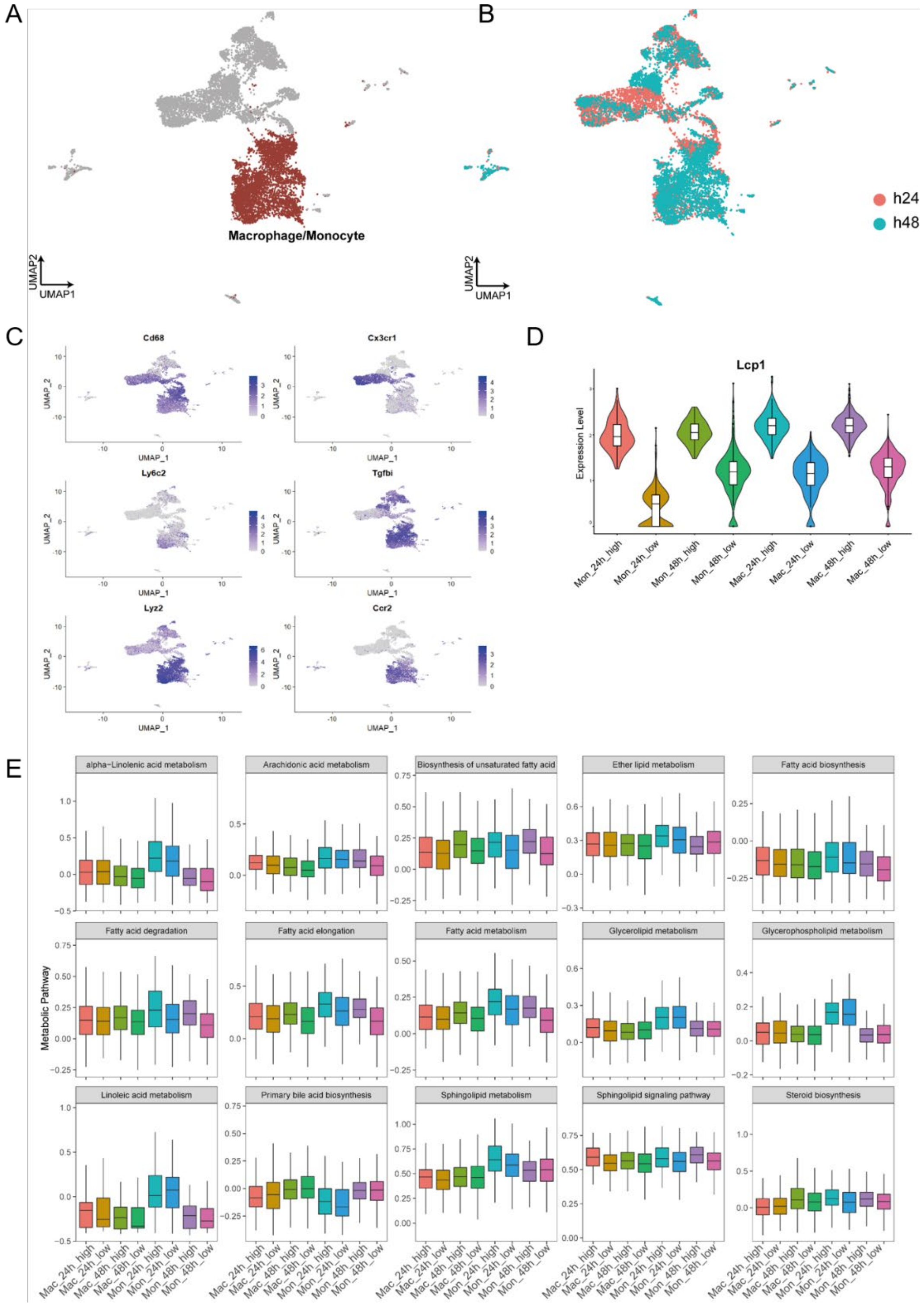

Figure S4. Heterogeneity in lipid metabolism of Lcp1^{high} and Lcp1^{low} macrophages and monocytes in post-stroke brains. (A-B) UMAP visualization illustrating clusters of cells isolated from ipsilateral (IL) hemispheres at 24 h and 48 h post-MCAo. (C) UMAP

representation highlighting specific markers for each identified cell cluster. (D) Categorization of $Lcp1^{high}$ and $Lcp1^{low}$ macrophages and monocytes at 24 h and 48 h post-tMCAO. (E) Box plots depicting expression levels related to lipid metabolism pathways in Lcp1^high and Lcp1^low macrophages and monocytes, in reference to Fig. 1D.
